# Applicability of methods for fluid responsiveness prediction in the ICU

**DOI:** 10.1186/cc10842

**Published:** 2012-03-20

**Authors:** P Mendes, L Miranda, M Park, L Azevedo, B Rodrigues, E Queiroz, G Schettino, L Taniguchi

**Affiliations:** 1 Hospital das Clinicas, São Paulo, Brazil; 2 Hospital Sirio Libanes, São Paulo, Brazil

## Introduction

Volume expansion is a frequent and widely used therapy in hemodynamically unstable patients but may lead to complications. To ensure appropriate indication of fluid administration, evaluation of fluid responsiveness by dynamic parameters is suggested, although it requires specific conditions not always present in ICU patients. The aim of this study was to analyze the applicability of parameters for evaluation of fluid responsiveness in the ICU.

## Methods

We conducted a prospective observational study in two ICUs. Volume expansions performed in ICU patients at the discretion of the physician in charge were analyzed for the presence of conditions that allowed adequate fluid responsiveness evaluation. The presence of central venous, pulmonary arterial or peripheral arterial catheters, invasive mechanical ventilation and ventilator settings, echocardiography availability, presence of arrhythmias, use of sedation and vasoactive drugs were registered. Percentages of patients who fulfilled conditions for dynamic parameters (such as pulse pressure variation, stroke volume variation and echocardiographic analysis) were recorded.

## Results

Ninety volume expansions in 68 patients were performed during the study period. Central venous catheter was present 58.9% of the time. In 41.1% of the cases patients were in spontaneous ventilation. No patients used a pulmonary artery catheter. An echocardiography machine with an attending physician trained for critical care echocardiography was available in 8.9%. An arterial catheter was available in 21% of the volume expansions and mechanical ventilation was present in 31.1% of the cases (67.3% of ventilated patients were using controlled mode of ventilation). The association of mechanical ventilation in controlled mode with an arterial catheter in place and no restrictions for performing analysis of dynamic parameters was present in only 7.7% of patients. Considering all dynamic parameters described here, the use of any method for predicting fluid responsiveness was possible in 15.6% of the volume expansions performed in our ICU.

## Conclusion

The use of dynamic parameters for predicting fluid responsiveness in the ICU may have restricted applicability since the necessary conditions are often not present.

